# The Hedgehog Receptor Patched1 in T Cells Is Dispensable for Adaptive Immunity in Mice

**DOI:** 10.1371/journal.pone.0061034

**Published:** 2013-04-08

**Authors:** Kai D. Michel, Anja Uhmann, Ralf Dressel, Jens van den Brandt, Heidi Hahn, Holger M. Reichardt

**Affiliations:** 1 Institute for Cellular and Molecular Immunology, University of Göttingen Medical School, Göttingen, Germany; 2 Institute for Human Genetics, University of Göttingen Medical School, Göttingen, Germany; Innsbruck Medical University, Austria

## Abstract

Hedgehog (Hh) signaling modulates T cell development and function but its exact role remains a matter of debate. To further address this issue we made use of conditional knock-out mice in which the Hh receptor Patched1 (Ptch) is inactivated in the T cell lineage. Thymocyte development was moderately compromised by the deletion of Ptch as characterized by reduced numbers of CD4 and CD8 single-positive cells. In contrast, peripheral T cells were not affected. Proliferation and IFNγ secretion by *Ptch*-deficient T cells were indistinguishable from controls irrespectively of whether we used strong or suboptimal conditions for stimulation. Analysis of CTL and T_reg_ cell functions did not reveal any differences between both genotypes, and T cell apoptosis induced by glucocorticoids or γ-irradiation was also similar. Surprisingly, absence of Ptch did not lead to an activation of canonic Hh signaling in peripheral T cells as indicated by unaltered expression levels of *Gli1* and *Gli2*. To test whether we could uncover any role of Ptch in T cells *in vivo* we subjected the mutant mice to three different disease models, namely allogeneic bone marrow transplantation mimicking graft-versus-host disease, allergic airway inflammation as a model of asthma and growth of adoptively transferred melanoma cells as a means to test tumor surveillance by the immune system. Nonetheless, we were neither able to demonstrate any difference in the disease courses nor in any pathogenic parameter in these three models of adaptive immunity. We therefore conclude that the Hh receptor Ptch is dispensable for T cell function *in vitro* as well as *in vivo*.

## Introduction

The Hh signaling pathway plays a critical role in development, cell fate decisions and tissue growth. Ptch, the receptor for Hh, inhibits its signaling partner Smoothened (Smo). Binding of Hh to Ptch or inactivating *Ptch* mutations result in derepression of Smo. This in turn triggers a cascade of downstream events which culminate in the activation of the Gli transcription factors Gli2 and Gli3, eventually leading to the expression of Hh target genes. Those include *Gli1*, which further amplifies the initial Hh signal at the transcriptional level, and frequently *Ptch* itself [1], [2].

Several members of the Hh signaling pathway such as Smo, Ptch and Gli1 are expressed in T cells [3], [4]. As a matter of fact, various experimental studies indicated that Hh signaling plays a crucial role in T cell development. For example, Sonic Hedgehog (Shh), the main mediator of Hh signaling, regulates differentiation from double-negative to double-positive thymocyte and controls thymocyte progenitor homeostasis [5], [6], [7]. In the thymus, cell-intrinsic Gli2 levels modulate the ratio of CD4 to CD8 single-positive cells [8], and stromal Gli3 expression was proposed to be involved in the differentiation of T cells [9]. In addition, we and others have identified Ptch as an exclusively T cell-extrinsic factor necessary for proper development of T cells at their prethymic stage [10], [11], [12].

Besides its involvement in T cell development, Hh signaling may also control the function of mature T lymphocytes. Analysis of peripheral T cells revealed that activation of CD4^+^ or CD8^+^ T cells with anti-CD3/CD28 antibodies increased the expression of *Smo* [3]. Addition of recombinant Shh-N enhanced the proliferative capacity of T cells, in particular under suboptimal conditions [3], [4], [13]. It also increased production of cytokines such as IL-2, IL-4, TNFα and IFNγ elicited by the treatment with anti-CD3 antibodies and led to an upregulation of activation markers such as CD25 and CD69 [3], [13]. Shh-N cooperated with anti-CD3 antibodies in enhancing cyclin A and cytokine-inducible SH2-containing protein (CIS-1) expression, thus mimicking T cell receptor costimulation [3]. In contrast, Shh-N did not augment Bcl-X_L_ levels in anti-CD3 stimulated CD4^+^ T cells, indicating that Hh and CD28 signaling share some but not all downstream targets [3]. A possible explanation for the impact of Hh signaling on T cell proliferation came from the finding that Bcl-2 is upregulated by addition of Shh during T cell activation [4].

On the other hand, several data argue for a repressive rather than an activating function of Hh signaling in T lymphocytes. Inhibition of the Hh pathway by transgenic overexpression of the repressor form of Gli2 under the lck promoter (Gli2ΔC2) increased differentiation from double- into single-positive thymocytes and augmented peripheral T cell numbers [14]. The Gli2ΔC2 transgene also conferred hyper-responsiveness when T cells were activated by ligation with anti-CD3 and anti-CD28 antibodies [14]. *Vice versa*, constitutive activation of Hh signaling by expression of a transgenic activator form of Gli2 under the control of the lck promoter (Gli2ΔN2) inhibited T cell activation and proliferation, probably by repressing TCR signal transduction [8]. Finally, one study in which Smo was conditionally deleted from T cells failed to reveal any influence of the loss of Hh signaling on anti-CD3 induced T cell proliferation [7]. This highlights that the currently available data regarding the role of Hh signaling in T cells are highly contradictory.

We recently reported that thymocyte development was independent of T cell-intrinsic Ptch expression [12]. Using a pure C57BL/6 background we here reinvestigated the role of Ptch in T cell development and additionally analyzed its function in peripheral T cells. This was accomplished by conditionally ablating Ptch by breeding *Ptch^flox/flox^* mice with *CD4Cre* transgenic mice, by analyzing thymocytes and various subsets of peripheral T lymphocytes *in vitro* and by subjecting the mutant mice to three different models of adaptive immune responses *in vivo*. However, despite a comprehensive set of assays addressing many different aspects of T cell function we were unable to identify any role of Ptch in this cell type. Our findings therefore argue that the Hh receptor Ptch, although not necessarily Hh signaling itself, does not play a major role in peripheral T cells.

## Materials and Methods

### Animal Experimentation

All mice were bred under SPF conditions in our animal facility in Göttingen and used at an age of 6–24 weeks. Food and drinking water were provided *ad libitum*. *Ptch^flox/flox^ CD4Cre^+/−^* mice were obtained by crossing *Ptch^flox/flox^* mice [11] with *CD4Cre^+/−^* transgenic mice [15], which results in the recombination of the *Ptch^flox^* locus starting at the DN3 stage of thymocyte development [15]. Importantly, a T cell-specific phenotype has neither been reported for *CD4Cre^+/−^* transgenic nor *Ptch^flox/flox^* mice, which allowed us to use them as controls in our experiments [7], [11], [15]. For most *in vitro* and all *in vivo* experiments mice had been backcrossed to the C57BL/6 background for more than 10 generations. Genotyping was achieved by PCR using the previously described primer combinations [12]. C3H/HeN, C57BL/6 and Balb/c mice were purchased from Charles River (Sulzfeld, Germany). All animal experiments were conducted according to ethical standards of humane animal care and approved by the authorities of Lower Saxony (*Nds. Landesamt für Verbraucherschutz und Lebensmittelsicherheit*, Permit Numbers: 33.9.42502-04/007/08; 33.9.42502-04/049/08; 33.14.42502-04/109/09). All efforts were made to minimize suffering of the mice.

### Flow Cytometry

Thymocytes and splenocytes were obtained by mechanical breakup of freshly dissected organs using forceps before the cells were passed through a 40 µm nylon mesh and washed in phosphate-buffered saline (PBS) plus 0.1% BSA. Alternatively, cells were directly used after magnetic separation or cell culture. All antibodies for FACS staining were obtained from BD Biosciences (Heidelberg, Germany), BioLegend (Uithoorn, The Netherlands), eBiosciences (Frankfurt, Germany) or AbD Serotech (Düsseldorf, Germany) and directed against the following antigens (clone name in parenthesis): TCRβ (H57-597), CD3ε (17A2), CD4 (RM4-5), CD8α (53-6.7), CD25 (7D4), CD44 (IM7), CD69 (H1-2F3), GITR (DTA-1), FoxP3 (FJK-16s), F4/80 (CI:A3-1), Ly-6C/G (Gr-1; RB6-8C5) and SiglecF (E50-2440). 7-AAD was purchased from BD Biosciences. The antibodies were either directly labeled with FITC, PE, PerCP, PE-Cy7, APC or APC-Cy7 or coupled to biotin, which was detected using a streptavidin-fluorochrome conjugate. Staining was performed according to standard procedures [16] and analyzed using a FACS Canto II device in combination with FACSDiva (BD Biosciences) or FlowJo (Treestar, Ashland/OR, USA) software. Intracellular staining of FoxP3 was accomplished using the *Foxp3/Transcription Factor Staining Buffer Set* according to the manufacturer’s instructions (eBioscience).

### T cell Purification

T cells were magnetically isolated from total splenocytes as previously described [17] by using the *Pan T Cell Isolation Kit II* in conjunction with an autoMACS separator (both from Miltenyi Biotech, Bergisch Gladbach, Germany). Cell purity was assessed by FACS analysis and was routinely around 95%. CD4^+^CD25^+^ T_reg_ cells and CD4^+^CD25^−^ Th cells used for suppression assays were purified by employing the *Regulatory T Cell Isolation Kit* together with an autoMACS separator as described elsewhere [18]. Cell purity was determined by FACS analysis using antibodies against TCRβ, CD4, GITR and FoxP3 and was routinely greater than 95%.

### RNA Isolation and Quantitative RT-PCR

Total RNA was isolated from purified splenic T cells using the *Quick-RNA Mini Prep Kit* (Zymo Research, Irvine, CA, USA) or from mouse embryos using TRIZOL reagent (Invitrogen, Carlsbad, CA, USA). Reverse transcription was achieved with the help of the *iScript cDNA Synthesis Kit* (Bio-Rad, München, Germany) according to the manufacturers’ instructions. For relative quantification of gene expression, qRT-PCR was performed using the 7500 Real Time PCR System in conjunction with the *Power SYBR Green PCR Master Mix* (both from Applied Biosystems). Detection of individual transcripts was achieved using the following primer combinations: *wt Ptch* (5′- AAA GCC GAA GTT GGC CAT GGG TAC -3′/5′- TGC TTG GGA GTC ATT AAC TGG A -3′), *Ptch^del^* (5′- AAA GCC GAA GTT GGC CAT GGG TAC -3′/5′- TTA AAC AGG CAT AGG CAA GCT GAC -3′), *Ptch2* (5′- TCC AAG TAT CAC TCT ATG GGA AAT C -3′/5′- TTC TCA ATC ATC CGC TCG AT -3′), *Gli1* (5′- TAC ATG CTG GTG GTG CAC ATG -3′/5′- ACC GAA GGT GCG TCT TGA GG -3′), *Gli2* (5′- GGT CAT CTA CGA GAC CAA CTG C -3′/5′- GTG TCT TCA GGT TCT CCA GGC -3′). Amplification of *HPRT1* (5′- GTC CTG TGG CCA TCT GCC TA -3′/5′ -GGG ACG CAG CAA CTG ACA TT -3′) served to normalize for the amount of cDNA in each sample. All samples were measured in duplicates and analyzed using the Sequence detection Software (Applied Biosystems).

### Apoptosis Assay

Apoptosis of splenic T cells was induced by treatment with dexamethasone (Dex) or exposure to γ-irradiation. In brief, 2×10^5^ cells were seeded in 96-well flat bottom plates and water-soluble Dex (Sigma-Aldrich, Taufkirchen, Germany) was added at escalating doses to the cultures. Alternatively, cells were exposed to different doses of γ-irradiation before culture using a RS 225 X-Ray Research System (Gulmay Medical Systems, Chertsey, Surrey, UK) operated at 150 kV, 15 mA and with a 0.5 mm Cu filtration. After 24–96 hours, cells were harvested, stained with 7-AAD and the amount of viable cells was determined by FACS analysis.

### T Cell Activation and Proliferation Assay

For polyclonal T cell activation, 10^5^ purified T lymphocytes were plated in 96-well flat bottom plates and stimulated for 24–72 hours either by adding ConA (Sigma-Aldrich) or soluble anti-CD3 and anti-CD28 antibodies (BD Biosciences or Biolegend) in suboptimal (0.5 µg/ml and 0.01 µg/ml, respectively) or optimal (2.5 µg/ml and 1.0 µg/ml, respectively) concentrations. Cells were stimulated in 200 µl RPMI 1640 medium with Glutamax, 10% fetal calf serum (FCS), 100 U/ml penicillin and 100 µg/ml streptomycin (all from Invitrogen, Karlsruhe, Germany). For quantification of IFNγ levels, an 50 µl aliquot of the supernatant was collected from each well and analyzed by ELISA using the BD OptEIA mouse IFNγ ELISA Set (BD Biosciences) according to the manufacturers’ instructions. For quantification of T cell proliferation, the cells were subsequently labeled with ^3^H-thymidine (Hartmann Analytics, Braunschweig, Germany) at a dose of 37 kBq/well and cultured for another 16 hours. The labeled DNA was collected onto Filtermat A glassfibre filters using a MicroBeta Filtermate-96 Harvester and encapsulated by MeltiLex solid scintillator. Quantification of the incorporated radioactivity was achieved using a MicroBeta^2^ ß-scintillation counter (all Perkin Elmer, Rodgau, Germany).

### Suppression Assay

The suppressive capacity of T_reg_ cells was determined essentially as described [18]. Conventional CD4^+^CD25^−^ Th cells (10^5^ cells/well) were cultured in RPMI 1640 medium with Glutamax supplemented with 10% FCS and antibiotics in 96-well U-bottom plates with different ratios of syngeneic CD4^+^CD25^+^ T_reg_ cells. Both cell types were either purified from *Ptch^flox/flox^* or *Ptch^flox/flox^* CD4Cre^+/−^ mice. Polyclonal activation was achieved by adding 1 µg/ml soluble anti-CD3 and 5 µg/ml anti-CD28 antibodies into the cultures. Th or T_reg_ cells alone (stimulated with anti-CD3 and anti-CD28) or unstimulated Th cells served as positive and negative controls. After 48 hours, supernatants were collected and IL-2 levels were assessed by using the BD OptEIA mouse IL-2 ELISA Set (BD Biosciences) according to the manufacturers’ instructions.

### Cytotoxicity Assay

Alloreactive CTLs were generated by intraperitoneal immunization of *Ptch^flox/flox^* and *Ptch^flox/flox^ CD4Cre^+/−^* mice (H2^b^) with 2×10^7^ splenocytes obtained from C3H/HeN (H2^k^) mice. The immunization was repeated twice with an interval of 10 days. Another 10 days later the splenocytes were harvested and restimulated *in vitro* for 5 days by co-culturing 7.5×10^5^ responder cells with 7.5×10^5^ irradiated (25 Gy) splenocytes from C3H/HeN (H2^k^) mice. Coculture was done in 96-well U-bottom plates in 200 µl of NaHCO_3_-buffered Dulbecco’s modified Eagle’s medium (DMEM, Biochrom, Berlin, Germany), supplemented with 10% FCS (Biochrom), 1 mM sodium pyruvate, 2 mM L-glutamine, 100 U/ml penicillin, 100 µg/ml streptomycin, 50 µM 2-mercaptoethanol (all from Sigma-Aldrich), 20 ng/ml recombinant mouse IL-2 (Immunotools, Friesoythe, Germany) and 50 µl of supernatant from ConA-stimulated rat lymphocytes. Mouse fibroblast Ltk^−^ cells (H2^k^) were used as target cells for the alloreactive CTLs in ^51^chromium release assays, which were performed as described previously [19].

### Measurement of Ig Serum Levels by ELISA

MaxiSorp flat bottom 96-well plates (Nunc GmbH, Langenselbold, Germany) were coated over night at 4°C with 50 µg/ml ovalbumin (Ova) in coating buffer (0.1 M sodium carbonate, pH = 9.5). The wells were washed with 0.05% Tween-20 in PBS and blocked with 10% FCS in PBS for 1 hour at room temperature. Subsequently, serum samples were added over night at 4°C. For IgE detection, the serum was initially incubated with Protein G PLUS agarose (Santa Cruz Biotechnology, Santa Cruz, CA, USA) to remove IgG antibodies, centrifuged and only the IgG-free supernatant was used. Ova-specific immunoglobulins were detected using HRP-coupled anti-IgG1, anti-IgG2a and anti-IgE specific antibodies (Southern Biotech, Birmingham, AL, USA). The reaction was quantified by measuring the absorbance at 450 nM and 570 nm using a PowerWave 340 ELISA reader (BioTek, Winooski, VT, USA).

### Histology

Fixed tissue samples were dehydrated, embedded in paraffin and cut into 5 µm slices. The sections were mounted onto slides and stained with hematoxylin and eosin (Merck, Darmstadt, Germany) according to standard protocols. From each mouse lung, several sections were taken from central and peripheral regions and microphotography was performed using an Olympus BX51 microscope with an Olympus ColorView Camera operated by analySIS^B^ software.

### Graft-versus-host Disease (GvHD) Mouse Model

To induce an acute GvHD reaction, 8–10 weeks old male Balb/c recipient mice received 10^7^ T cell-depleted syngeneic bone marrow cells plus 2×10^6^ T cells from *Ptch^flox/flox^ CD4Cre^+/−^* or *Ptch^flox/flox^* control donor mice one day after irradiation with 8.5 Gy. In detail, bone marrow was isolated from femurs of C57Bl/6 mice and T cell depletion was achieved using anti-CD90.2 microbeads and an autoMACS separator (both from Miltenyi Biotech) according to the manufacturer’s instructions. Cell purity was assessed by FACS and revealed that T cell contamination was routinely less than 1%. T cells were isolated from spleens and cervical, mesenteric and inguinal lymph nodes of *Ptch^flox/flox^ CD4Cre^+/−^* and *Ptch^flox/flox^* control C57BL/6 mice as described before [17]. Bone marrow with or without (control) T cells was mixed and injected in a total volume of 200 µl PBS into the tail vein. Starting from one day before cell transfer, mice were kept on antibiotic water (25 µg/ml neomycin) for four weeks. Mice were monitored every other day for survival and their health status was assessed according to five clinical parameters (posture, activity, fur ruffling, diarrhea and weight loss) as described [17], each of which received a score from 0 to 2, resulting in a total score between 0 and 10. Due to ethical reasons, mice were sacrificed when the total clinical score exceeded a value of 7 for more than one day.

### Allergic Airway Inflammation Mouse Model

Female *Ptch^flox/flox^* and *Ptch^flox/flox^ CD4Cre^+/−^* mice were immunized on days 0, 9 and 18 by intraperitoneal injections of 10 µg chicken egg ovalbumin (Ova grade V; Sigma-Aldrich) together with 1.5 mg of aluminum hydroxide (Alhydrogel 2%, Invivogen, San Diego, Ca, USA) as the adjuvant in a total volume of 150 µl PBS. Control mice received intraperitoneal injections without Ova. From days 28 to 30, both immunized and control mice were challenged by daily intranasal administration of 150 µg Ova solubilized in PBS. Analysis was performed on day 32 after the initial immunization. To this end, mice were sacrificed by CO_2_ inhalation and a midline neck incision was made to cannulate the trachea. Lungs were rinsed with 500 µl PBS and the recovered BALF cell suspension was centrifuged, counted with a Neubauer counting chamber and used for FACS analysis. Eosinophils were distinguished from neutrophils and macrophages based on the combined staining for Siglec-F, F4/80 and Gr-1 (details are available upon request). Lungs were incubated at room temperature in Roti-Histofix 4% (Carl Roth GmbH, Karlsruhe, Germany) over night before they were used for histological analysis. For serum analysis, blood was collected from the vena renalis and centrifuged; the serum was then collected and frozen for later analysis. Splenocytes were isolated as described above and erythrocytes were lysed using a haemolysis buffer (400 µM Tris, 155 mM ammonium chloride, pH = 7.2). The cells were plated in 96-well flat bottom plates and stimulated with 10 µg/ml Ova for 72 hours. Non-stimulated or ConA-stimulated cells served as controls. Proliferation and cytokine production were assessed as described above.

### Melanoma Mouse Model

The previously published B16-F10 murine melanoma cells were kindly provided by Dr. Jürgen Becker (Clinic of Dermatology, University of Würzburg, Germany) [20]. The cells were grown in RPMI 1640 supplemented with 10% FCS, 100 U/ml penicillin and 100 µg/ml streptomycin. For tumor vaccination, cells were harvested, washed twice in PBS, irradiated with 125 Gy and mixed at a ratio of two volumes of cells to one volume of complete Freund’s adjuvant (CFA; Sigma-Aldrich). Subsequently, 10^6^ cells were injected in a total volume of 150 µl subcutaneously into the left flank. Ten days after immunisation, mice were challenged with 10^4^ live B16-F10 cells into the right flank. Starting from day 7 after challenge, mice were regularly monitored for palpable tumors. Tumor size was recorded from two caliper measurements of the longest (a) and shortest (b) diameter and the tumor volume was calculated according to the following formula: volume = a×b^2^×0.4 [21]. The experiment was terminated for individual mice when the tumor volume exceeded 800 mm^3^ or when ulceration or bleeding occurred.

### Statistical Analysis

Statistical analysis was performed by unpaired t-test, Mann Whitney or log-rank test, and the data are depicted as mean ± SEM. To analyze the cytotoxicity assay a 2-way ANOVA test was employed. GraphPad Prism® software was used in all cases.

## Results

### Disruption of Ptch in the T Cell Lineage Moderately Impacts Thymocyte Development while Peripheral T Lymphocytes are Unaffected

**Figure 1:**
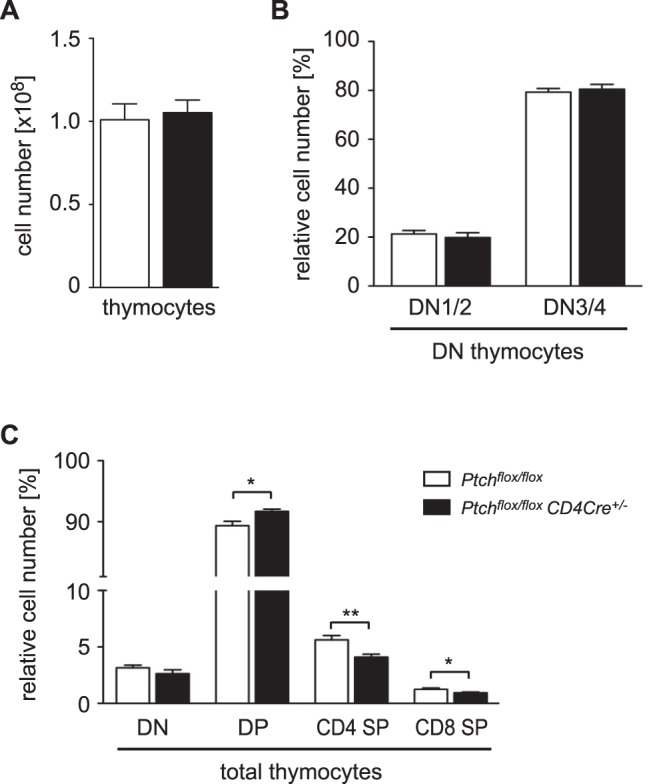
Flow cytometric analysis of thymocyte development. Thymocytes were isolated from *Ptch^flox/flox^* control or *Ptch^flox/flox^ CD4Cre^+/−^* mutant mice, counted and stained for CD4, CD8, and CD44 to identify different developmental stages. (A) Mean number of total thymocytes ± SEM is shown for four mice of each genotype. (B) Percentages of CD4^−^CD8^−^CD44^+^ (DN stages 1 and 2) or CD4^−^CD8^−^CD44^−^ (DN stages 3 and 4) thymocytes amongst total DN thymocytes are depicted. (C) Percentages of DN, DP and CD4 SP or CD8 SP cells amongst total thymocytes are shown. Panels B and C refer to the mean ± SEM of four mice of each genotype. Statistical analysis was performed by unpaired t-test (*: p<0.05). Differences were not statistically different unless otherwise indicated.

We previously reported that T cell-specific deletion of Ptch did not impact thymocyte development in *Ptch^flox/flox^ CD4Cre^+/−^* mice [12]. As our findings were in contrast to several other reports describing an influence of Hh signaling on the transition from the double-positive (DP) to the single-positive (SP) stage of thymocyte development [22], [23], [24], we decided to repeat the analyses with mice backcrossed to the C57BL/6 background. Although the overall thymic cellularity (Figure 1A) and the percentages of double-negative (DN) thymocytes (Figure 1B,C) were similar in both genotypes, we found that the relative number of DP thymocytes was increased in *Ptch^flox/flox^ CD4Cre^+/−^* mutant mice while the percentages of CD4 and CD8 SP thymocytes were decreased (Figure 1C). Even though the magnitude of these differences was small, they nevertheless reached statistical significance and were qualitatively in line with previous reports [22], [23], [24]. This encouraged us to ask whether the deletion of Ptch would also affect peripheral T cells.

**Figure 2:**
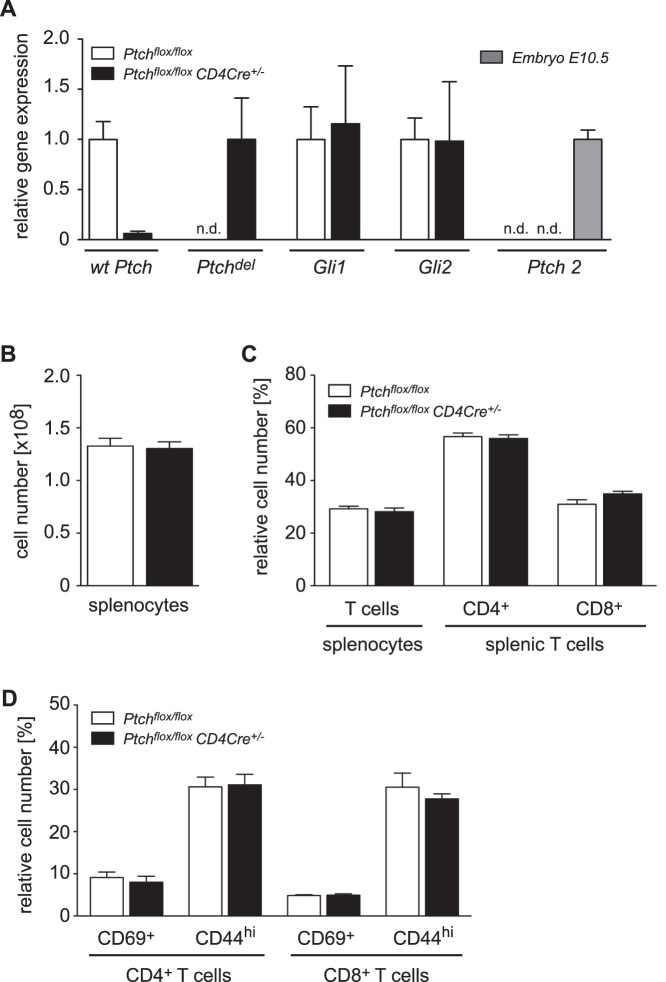
Gene expression and flow cytometric analysis of peripheral T cells. Splenic T cells were purified from *Ptch^flox/flox^* control or *Ptch^flox/flox^ CD4Cre^+/−^* mutant mice and used to generate cDNA for qRT-PCR analysis. Alternatively, total splenocytes were isolated from mice of each genotype and stained for TCRβ, CD4, CD8, CD44 and CD69 to identify different peripheral T cell subpopulations. (A) Relative expression of *wt Ptch* transcripts derived from the *Ptch^flox^* allele, *Ptch^del^* transcripts after Cre-mediated recombination of the Ptch^flox^ allele, *Gli1* and *Gli2* transcripts as well as *Ptch2* transcripts are depicted. As a positive control for *Ptch2* expression, cDNA from wt mouse embryos at stage E10.5 was used for PCR amplification. Gene expression was normalized to *HPRT1* and the transcriptional level of each gene in *Ptch^flox/flox^* control mice or E10.5 embryos, respectively, was set to 1.0. All panels show the mean ± SEM of five *Ptch^flox/flox^* and three *Ptch^flox/flox^ CD4Cre^+/−^* mice; n.d.: not detectable. Measurement of all samples was performed in duplicates. (B) Mean number of total splenocytes ± SEM is shown for ten *Ptch^flox/flox^* and eight *Ptch^flox/flox^ CD4Cre^+/−^* mice. (C) The percentages of T cells amongst all splenocytes and of CD4^+^ or CD8^+^ T cells amongst total splenic T cells is depicted. (D) T cells showing a recently activated (CD69^+^) or effector memory (CD44^hi^) phenotype were identified amongst the CD4^+^ and CD8^+^ splenic T cells. Panels C and D show the mean ± SEM of five *Ptch^flox/flox^* and seven *Ptch^flox/flox^ CD4Cre^+/−^* mice. Statistical analysis was performed by unpaired t-test (*: p<0.05).

In our previous work we demonstrated that recombination of the *Ptch* locus and the resulting expression of the non-functional *Ptch^del^* allele were almost complete in thymocytes of *Ptch^flox/flox^ CD4Cre^+/−^* mice [12]. Nonetheless, this did not lead to an upregulation of the two target genes *Gli1* and *Gli2* as one would have predicted for activated canonical Hh signaling [12]. Therefore we performed the same analysis for peripheral T cells by isolating splenocytes from mice of both genotypes followed by qRT-PCR analysis. Surprisingly, we again found that disruption of Ptch was almost complete in mutant T cells and that this had no effect on *Gli1* and *Gli2* expression (Figure 2A). We also analyzed expression of *Ptch2* to determine whether this highly homologous protein might compensate for the loss of Ptch. Importantly, we could not detect any *Ptch2* mRNA in T cells of either genotype while *Ptch2* was abundantly expressed at embryonic stage E10.5.

Next we studied the cellular composition of peripheral lymphoid organs. Size and cellularity of the spleen were unaltered in *Ptch^flox/flox^ CD4Cre^+/−^* mutant mice and the same was true for the percentages of splenic T cells as well as of CD4^+^ and CD8^+^ cells amongst them (Figure 2B,C). Similar observations were made for lymph nodes and peripheral blood (data not shown). To unravel a potentially more subtle impact of the ablation of Ptch, we enumerated recently activated T cells based on CD69 surface expression as well as the CD44^hi^ memory T cells. Nonetheless, the percentages of both populations were similar amongst the CD4^+^ or CD8^+^ T cells in *Ptch^flox/flox^* control and *Ptch^flox/flox^ CD4Cre^+/−^* mutant mice irrespective of whether we analyzed spleen, lymph nodes or blood (Figure 2D and data not shown). We conclude that the deletion of Ptch in the T cell compartment does not impact the composition of peripheral T cells.

### Ablation of Ptch does not Impact Polyclonal T Cell Activation

**Figure 3:**
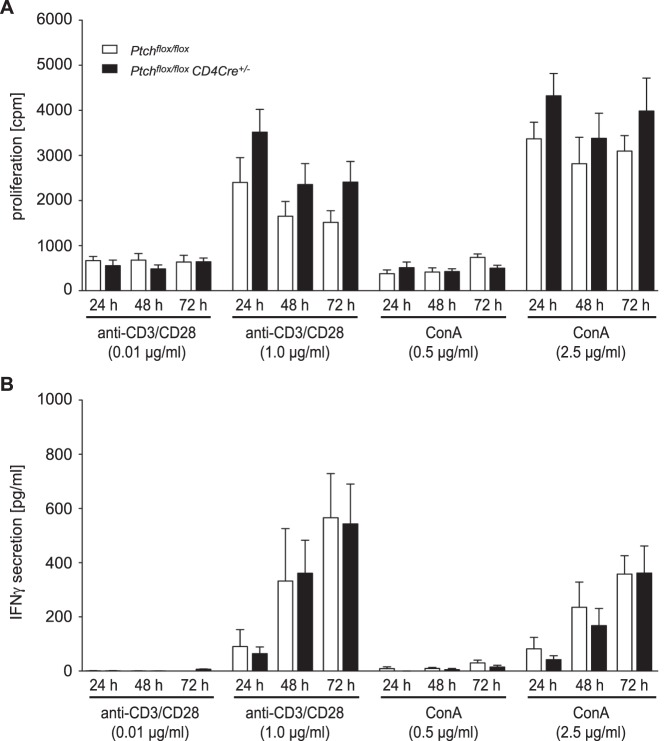
Proliferation and IFNγ production by activated T cells. Total T cells were isolated from the spleens of *Ptch^flox/flox^* control or *Ptch^flox/flox^ CD4Cre^+/−^* mutant mice and 10^5^ cells per well were stimulated with different concentrations of ConA or anti-CD3/CD28 antibodies in a total volume of 200 µl medium. For detection of IFNγ, 50 µl medium were removed at each time point and used for analysis by ELISA. For measurement of proliferation, an equal volume of fresh medium containing 37 kBq ^3^H-thymidine was added to the same wells and incubated for another 16 hours. (A) Proliferation was assessed by scintillation counting. (B) Quantification of IFNγ levels was achieved by ELISA. Each panel shows the mean ± SEM of five individual experiments. Statistical analysis for each experimental condition was performed by unpaired t-test and no differences were found in any case.

To explore the role of Ptch for T cell activation, we sorted splenic T lymphocytes and stimulated them either with anti-CD3/CD28 antibodies or Concanavalin A (ConA). Since Hh signaling was previously reported to be particularly relevant under suboptimal stimulation conditions, anti-CD3/CD28 and ConA were used at two different concentrations. T cell proliferation and IFNγ production were monitored over a 72 hours period by ^3^H-thymidine incorporation assay and ELISA, respectively. When we stimulated the T cells under optimal conditions (1.0 µg/ml anti-CD3/CD28 or 2.5 µg/ml ConA), proliferation of *Ptch^flox/flox^* control and *Ptch^flox/flox^ CD4Cre^+/−^* cells was strong but similar at any time point (Figure 3A). When we used anti-CD3/CD28 or ConA at suboptimal concentrations (0.01 µg/ml and 0.5 µg/ml, respectively), proliferation was overall much weaker but ablation of Ptch had again no effect (Figure 3A). Unlike activated T cells, resting T lymphocytes from control and *Ptch*-deficient mice hardly proliferated at all (data not shown). IFNγ secretion increased over time under optimal stimulation conditions, while it was very low under suboptimal conditions. Nonetheless, there was no difference between both genotypes (Figure 3B). We conclude that T cell activation is unaffected by the absence of Ptch both under optimal and suboptimal stimulation conditions.

### Ptch has No Impact on the Cytolytic Capacity of CTLs

**Figure 4:**
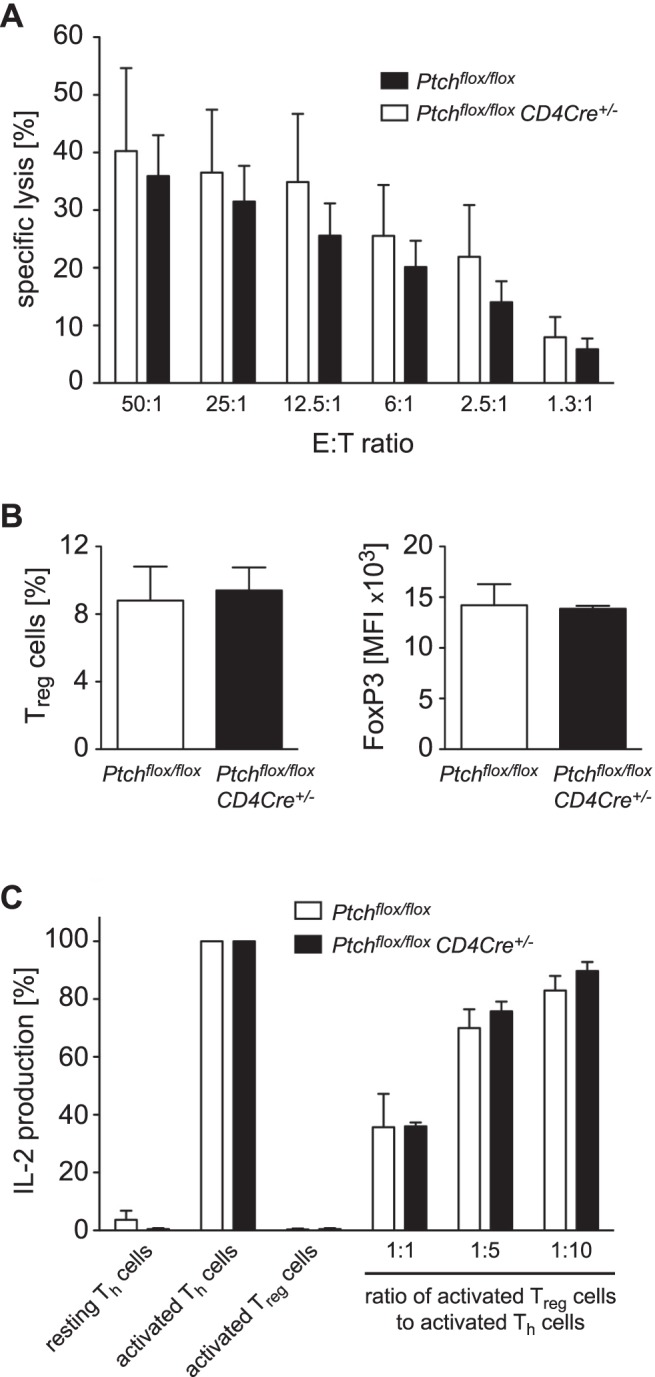
Cytolytic capacity of CTLs and suppressive activity of T_reg_ cells. (A) Specific lysis of Ltk^−^ target cells (H2^k^) by alloreactive CTLs derived from *Ptch^flox/flox^* control or *Ptch^flox/flox^ CD4Cre^+/−^* mutant mice (H2^b^), which had been immunized with splenocytes from C3H/HeN mice (H2^k^). Means of specific lysis of triplicates of the Ltk^−^ cells were determined for eight individual mice of both genotypes. The diagram shows the mean of specific lysis ± SEM for both genotypes at several effector to target (E:T) ratios. Statistical analysis was achieved by 2-way ANOVA (p = 0.216). (B) Abundance and FoxP3 expression of splenic TCRβ^+^CD4^+^CD25^+^GITR^+^FoxP3^+^ T_reg_ cells. T_reg_ cells in the spleen were enumerated by FACS analysis and their percentage amongst the CD4^+^ T cells is depicted as the mean ± SEM from three *Ptch^flox/flox^* and five *Ptch^flox/flox^ CD4Cre^+/−^* mice (left panel). The levels of intracellular FoxP3 expression in T_reg_ cells were quantified by FACS and the mean fluorescent intensity (MFI) is depicted as mean ± SEM for three *Ptch^flox/flox^* and four *Ptch^flox/flox^ CD4Cre^+/−^* mice (right). Both parameters were statistically similar based on analysis by unpaired t-test (C) Suppressive capacity of T_reg_ cells from *Ptch*-deficient and control mice. CD4^+^CD25^+^ T_reg_ cells were cocultured with CD4^+^CD25^−^ Th cells at ratios of 1∶1, 1∶5 and 1∶10. Polyclonal activation was achieved using anti-CD3/CD28 antibodies and IL-2 levels were assessed in the cell culture supernatants after 48 hours. All values are normalized to cultures of activated CD4^+^CD25^−^ Th cells which served as a positive control. Resting CD4^+^CD25^−^ Th cells and activated CD4^+^CD25^+^ T_reg_ cells served as negative controls. The figure shows the mean ± SEM of three (*Ptch^flox/flox^*) and four (*Ptch^flox/flox^ CD4Cre^+/−^*) independent experiments, respectively. Based on the analysis by unpaired t-test, the suppressive capacity of T_reg_ cells from both genotypes was similar.

We also determined the role of Ptch for the effector function of CD8^+^ T cells by analyzing the cytolytic capacity of alloreactive CTLs (Figure 4A). *Ptch^flox/flox^* control and *Ptch^flox/flox^ CD4Cre^+/−^* mutant mice (H2^b^) were repeatedly immunized with allogeneic splenocytes from C3H/HeN (H2^k^) mice. Subsequently, the splenocytes of the recipients were harvested, restimulated *in vitro* with splenocytes from C3H/HeN mice and subjected to a chromium release assay using Ltk^−^ target cells (H2^k^). The specific lysis of these targets cells by mutant and control CTLs was similar (p = 0.216 by 2-way ANOVA), indicating that Ptch was not required for the cytolytic function of CTLs *in vitro*.

### The Abundance and Suppressive Capacity of T_reg_ Cells is Independent of Ptch

To determine the abundance of naturally occurring T_reg_ cells in the spleen we performed a flow cytometric analysis. Approximately 8% of all CD4^+^ T cells in *Ptch^flox/flox^* control mice were CD25^+^GITR^+^FoxP3^+^ T_reg_ cells and their frequency was similar in mutant *Ptch^flox/flox^ CD4Cre^+/−^* mice (Figure 4B, left panel). Given the importance of the transcription factor FoxP3 for T_reg_ cell function, we determined its expression level in TCRβ^+^CD4^+^CD25^+^GITR^+^FoxP3^+^ cells by flow cytometry. However, there was no difference in FoxP3 expression between both genotypes (Figure 4B, right panel). The most important criterion to assess the function of T_reg_ cells is their capacity to suppress IL-2 secretion and proliferation of conventional CD4^+^ Th cells. Therefore, we magnetically purified CD4^+^CD25^−^ Th cells from mice of both genotypes and activated them with anti-CD3/CD28 antibodies. These cells were then used as indicator cells in co-cultures with CD4^+^CD25^+^ T_reg_ cells sorted either from *Ptch^flox/flox^* control or *Ptch^flox/flox^ CD4Cre^+/−^* mice. As revealed on the basis of IL-2 levels in culture supernatants, T_reg_ cells suppressed the function of conventional T cells in a dose-dependent manner. However, the suppressive capacity of T_reg_ cells was independent of Ptch expression (Figure 4C).

### Deletion of Ptch has No Impact on T Cell Apoptosis

**Figure 5:**
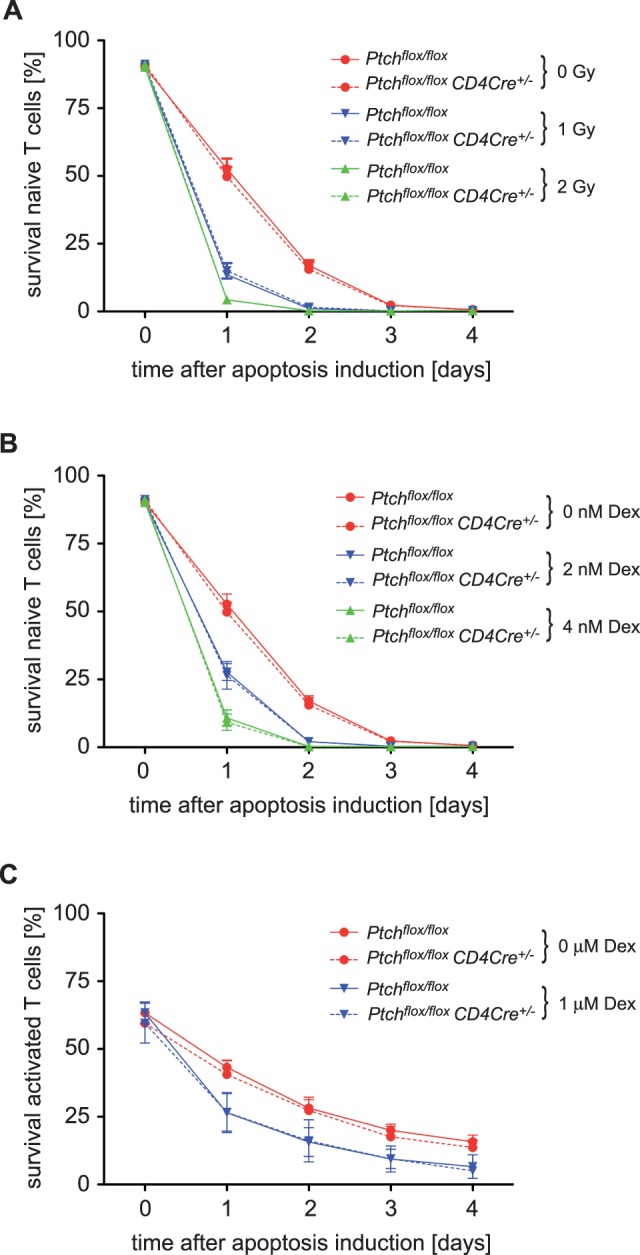
Sensitivity of T cells to apoptosis induction. (A) Apoptosis induction by γ-irradiation was assessed by culturing 2 x 10^5^ T cells following exposure to a dose of 1 or 2 Gy or without any manipulation. Cell viability was assessed every 24 hours by FACS analysis using 7-AAD. Results represent the mean ± SEM of three independent experiments. (B) Glucocorticoid-induced apoptosis was investigated by culturing 2 x 10^5^ splenic T cells from *Ptch^flox/flox^* control or *Ptch^flox/flox^ CD4Cre^+/−^* mutant mice in medium with or without 2 or 6 nM of water-soluble Dex for 4 days. Cell survival was determined as described above. Results represent the mean ± SEM of three independent experiments. (C) T cells were pre-activated with 1.5 µg/ml soluble anti-CD3 and anti-CD28 for 24 hours. Apoptosis was then induced by adding 1 µM of water-soluble Dex and cell viability was assessed up to four days after apoptosis induction similar as above. Results represent the mean ± SEM from three *Ptch^flox/flox^* or four *Ptch^flox/flox^ CD4Cre^+/−^* animals. Based on the analysis by unpaired t-test, apoptosis was not different between both genotypes.

Synthetic glucocorticoids such as dexamethasone (Dex) and DNA-damage caused by γ-irradiation are known to induce T cell apoptosis by activating the pro-apoptotic proteins Bim and Puma [25]. To assess the role of Ptch in this process, we sorted T cells from the spleens of *Ptch^flox/flox^* control or *Ptch^flox/flox^ CD4Cre^+/−^* mutant mice. Spontaneous cell death was indistinguishable between both genotypes. When T cells were γ-irradiated with a dose of 1 or 2 Gy and cultured for up to 4 days, we observed a dose- and time-dependent increase of cell death which was indistinguishable between both genotypes (Figure 5A). In another setting, we cultured T cells in the presence of either 2 nM or 6 nM Dex for up to 4 days. There was a dose- and time-dependent increase of apoptosis which was again unaltered in *Ptch*-deficient cells (Figure 5B). We also tested whether activated T cells may exhibit any difference concerning their sensitivity to apoptosis. Following activation by anti-CD3/CD28 antibodies for 24 hours the T cells were much more resistant to Dex-induced apoptosis as compared to naïve T cells, and not affected by the treatment unless the dose was considerably increased (Figure 5B,C). Nonetheless, activated T cells still underwent apoptosis in the presence of 10^−6 ^M Dex, but there was no difference between cells isolated from *Ptch^flox/flox^* control or *Ptch^flox/flox^ CD4Cre^+/−^* mice in this respect (Figure 5C). We conclude that ablation of Ptch does not impact apoptosis sensitivity, neither of resting nor activated peripheral T cells.

### Adaptive Immune Responses do not Require Ptch Expression in T Lymphocytes

**Figure 6:**
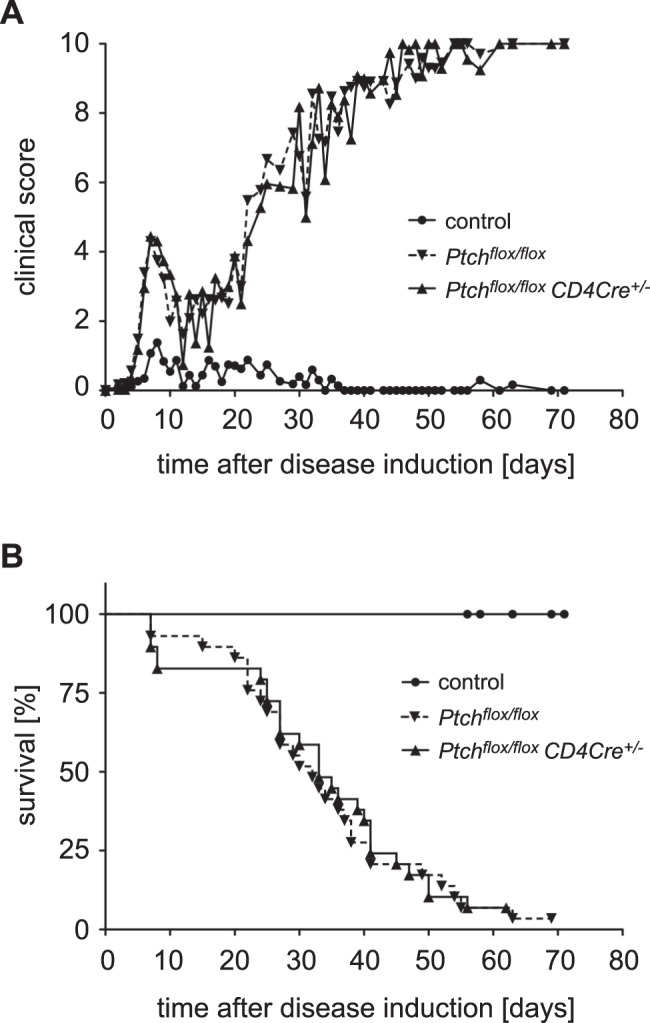
Morbidity and mortality after GvHD induction. GvHD was induced in irradiated Balb/c mice (8.5 Gy) by transferring 10^7^ T cell-depleted syngeneic bone marrow cells plus 2×10^6^ T cells from either *Ptch^flox/flox^* control or *Ptch^flox/flox^ CD4Cre^+/−^* mutant C57BL/6 donor mice (n = 29 for each genotype). Control animals received T cell depleted bone marrow only (n = 13). Mice were monitored every other day for clinical symptoms (A) and survival (B). The figures show the combined data of five independent experiments. Statistical analysis of the disease courses was performed by Mann Whitney test; in the case of the survival curves the log-rank test was employed. In both cases, no statistical significant difference between both genotypes was found.

Since we had not observed any major difference in T cell function *in vitro*, we wondered whether Ptch deletion might only be relevant under more physiological conditions *in vivo*. To address this question, we initially analyzed a mouse model of graft-versus-host disease (GvHD), which depends on the function of both Th cells and CTLs. In humans, GvHD occurs after transplantation of a MHC mismatched bone marrow graft containing mature alloreactive T cells. In our experimental model, lethally irradiated Balb/c mice were transplanted with bone marrow and purified T cells from either *Ptch^flox/flox^* control or *Ptch^flox/flox^ CD4Cre^+/−^* C57BL/6 mice. Upon encountering the allogeneic MHC molecules, the transferred T cells attack the host cells and induce a strong adaptive immune response dominated by Th1 cells. This leads to the occurrence of several typical disease symptoms, and eventually the mice succumb to death. Importantly, disease severity and mortality were similar in mice of both genotypes as confirmed by statistical analysis (Figure 6A,B). This indicates that Ptch in T cells does not affect any T cell effector functions required for the occurrence of GvHD.

**Figure 7:**
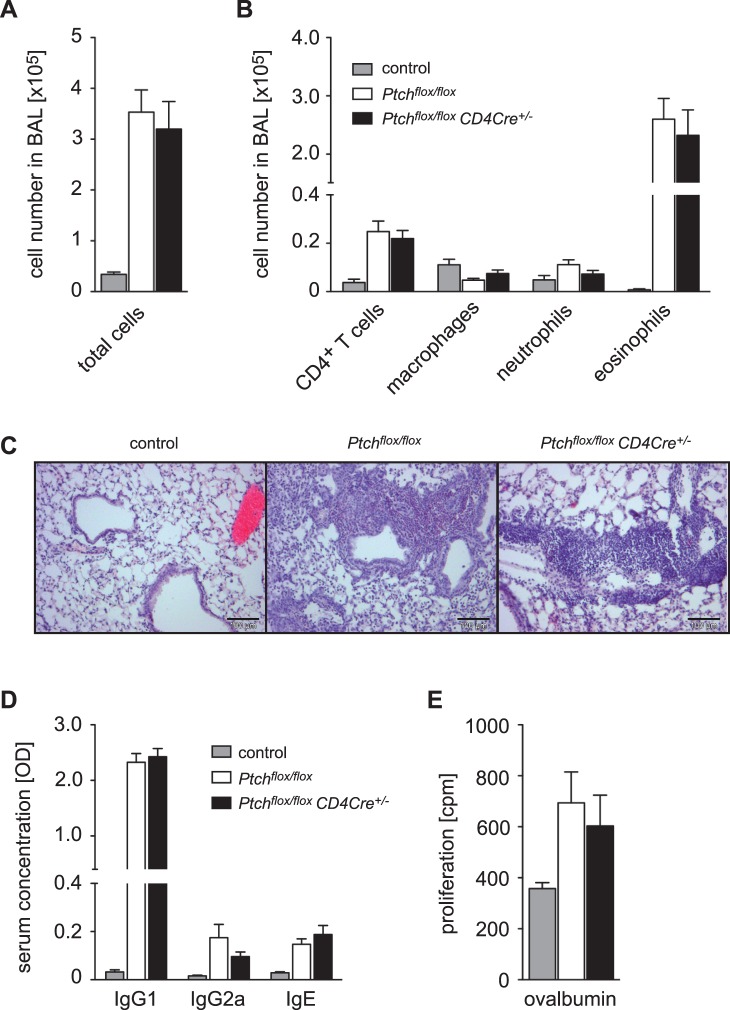
Lung infiltration, antibody production and T cell function after induction of allergic airway inflammation. *Ptch^flox/flox^* control and *Ptch^flox/flox^ CD4Cre^+/−^* mutant C57BL/6 mice were sensitized against Ova by repeated intraperitoneal injection of antigen plus adjuvant. Control mice (*Ptch^flox/flox^*) received adjuvant without Ova. Antigen challenge was achieved by intranasal delivery of dissolved Ova for three consecutive days and analysis was performed after a two day resting phase. (A) Lungs were washed *in situ* and total cell counts in the broncheoalveolar lavage fluid (BALF) were determined using light microscopy. (B) Identification of different populations of lung-infiltrating cells within the BALF by using FACS. (C) Lung histology of mice 48 hours after the last challenge. A control lung obtained from a non-sensitized mouse challenged with Ova is shown along with lungs of *Ptch^flox/flox^* and *Ptch^flox/flox^ CD4Cre^+/−^* mice which were both sensitized and challenged with antigen. No pathohistological signs of inflammation could be detected in control mice whereas a clear and massive cell influx was seen in sensitized and challenged mice. (D) Serum concentrations of Ova-specific IgG1, IgG2a and IgE immunoglobulins were quantified using ELISA and are depicted in the form of optical densities (OD). (E) Splenocytes were isolated and restimulated *ex vivo* with 10 µg/ml Ova for 72 hours and proliferation was assessed by ^3^H-thymidine incorporation assay. Results represent the mean ± SEM from nine non-sensitized control mice as well as thirteen *Ptch^flox/flox^* and fourteen *Ptch^flox/flox^ CD4Cre^+/−^* animals, respectively. Statistical analysis of all parameters was performed by unpaired t-test and no differences were found in any case.

In another approach we studied allergic airway inflammation, which is an experimental model of human asthma and represents a prototypic Th2 immune response. *Ptch^flox/flox^* control and *Ptch^flox/flox^ CD4Cre^+/−^* mice were repeatedly immunized with ovalbumin in adjuvant and subsequently challenged intranasally to induce an inflammatory response in the lung. Compared to control mice, intranasal ovalbumin application led to a strong infiltration of leukocytes into the lung (Figure 7A,C). The bronchoalveolar lavage fluid (BALF) was dominated by eosinophils but also contained CD4^+^ T cells, macrophages and neutrophils (Figure 7B). Nonetheless, neither the absolute numbers of the infiltrating cells nor their relative percentages were significantly different between both genotypes (Figure 7A–C). We also checked the titers of ovalbumin-specific antibody isotypes. The predominant one found in the serum was IgG1 with smaller amounts of IgG2a and IgE (Figure 7D), a finding which is typical for an allergic immune response induced in C57BL/6 mice. However, the antibody titers of all three isotypes were not significantly different between *Ptch^flox/flox^* control and *Ptch^flox/flox^ CD4Cre^+/−^* mice. The proliferative response of splenic T cells restimulated with ovalbumin *in vitro* was increased in immunized mice but to a similar extent in both genotypes (Figure 7E). Thus, we did not find any indication that the allergic immune response in a model of asthma was influenced by the presence of Ptch in T cells.

### The Tumor Surveillance Capacity of the Immune System is Unaffected by Ptch Ablation in T Cells

**Figure 8:**
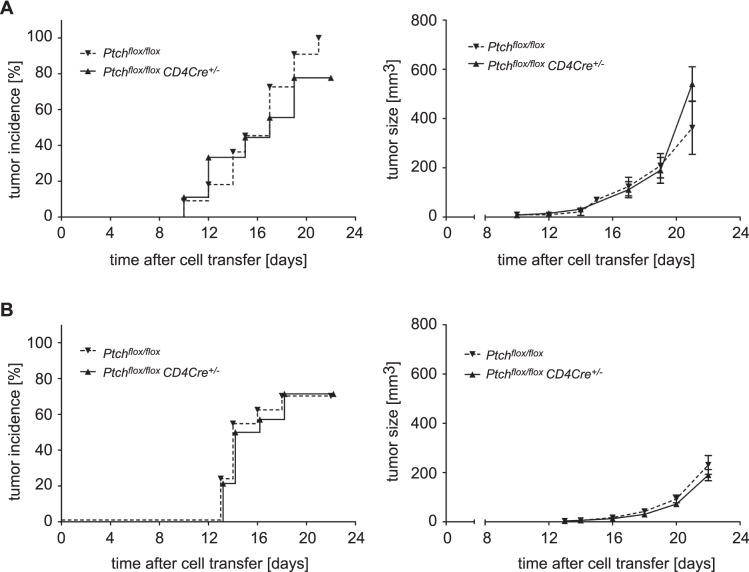
Tumor incidence and size after inoculation of B16 melanoma cells. C57BL/6 mice were inoculated with 10^4^ B16-F10 melanoma cells into the right flank and tumor growth was monitored over a period of three weeks. The incidence of palpable tumors (left panel) and the mean tumor size ± SEM (right panel) are depicted for both experimental setups. (A) Results of tumor incidence and size are shown for eleven *Ptch^flox/flox^* control and nine *Ptch^flox/flox^ CD4Cre^+/−^* mutant C57BL/6 mice in which tumorigenesis had been induced without additional manipulation. (B) To generate protective immunity, C57BL/6 mice were vaccinated with 10^6^ irradiated B16-F10 cells together with adjuvant subcutaneously into the left flank. Ten days later the vaccinated mice were challenged with 10^4^ viable B16-F10 cells subcutaneously into the right flank. Tumor incidence rate and mean tumor size ± SEM are shown for fourteen *Ptch^flox/flox^* and *Ptch^flox/flox^ CD4Cre^+/−^* mice each. Statistical analysis of tumor incidence and size was performed by Mann Whitney test and not found to be different between both genotypes.

Although we had not observed any effect of Ptch deletion on CTL and T_reg_ cell function, we considered it possible that a potential role in these T cell subpopulations might only become evident *in vivo*. One function of the immune system in which both cell types are important is tumor surveillance. Whereas CTLs are one of the major effector cell types responsible for the lysis of neoplastic cells, T_reg_ cells are known to be tumor promoting. To address this issue, we subcutaneously inoculated *Ptch^flox/flox^* control and *Ptch^flox/flox^ CD4Cre^+/−^* mice with B16-F10 melanoma cells and monitored tumor incidence and size over a four weeks period. However, we did not detect any differences neither in tumor progression nor size (Figure 8A). We also immunized the mice prior to tumor inoculation by using inactivated tumor cells aiming at eliciting a protective anti-tumor response. This procedure indeed decreased tumor incidence, time of onset and tumor size, but to the same extent in both genotypes as revealed by statistical analysis (Figure 8B). We therefore conclude that Ptch in T cells is dispensable for proper tumor surveillance by the adaptive immune system.

## Discussion

Stromal expression of the Hh receptor Ptch plays a critical role in pre-thymic T cell development [11], [12], yet it is unknown whether it also fulfills an intrinsic function in peripheral T cells. In fact, all major components of the Hh signaling pathway are expressed in T lymphocytes and, according to the current view, at least Hh and Gli2 are required for proper T cell function. Our current data support a role of the Hh receptor Ptch in intermediate and late thymocyte development but argue against an important function in peripheral T cells both *in vitro* and *in vivo*.

We employed a mouse model in which Ptch was specifically inactivated in T cells by Cre recombinase expressed under the control of the CD4 promoter, which becomes active at the DN3 stage of thymocyte development [15]. Using this strategy we found that Ptch inactivation, despite being highly efficient, affected thymocyte development only moderately. This finding was distinct from our earlier data showing that T cell development was completely independent of T cell-intrinsic Ptch expression [12]. The discrepancy could be explained by the different genetic backgrounds of the mouse strains used in both studies. Here we almost exclusively analyzed mice backcrossed to C57BL/6 for more than 10 generations, while we had previously used mice on a mixed Balb/c and C57BL/6 background.

The alterations in thymocyte development that we observed here were qualitatively similar to those reported to occur in response to activated Hh signaling [22], [23], [24]. Importantly, however, our analyses did not reveal any impact of the deletion of Ptch on the number and phenotype of peripheral T cells. *In vitro* analysis demonstrated that functional characteristics of conventional T cells such as proliferation and cytokine production in response to polyclonal stimulation were nearly unaltered in Ptch-deficient T cells. Of note, the proliferation rate of polyclonally activated Ptch deficient T cells was slightly higher as compared to control cells, but this difference only emerged under highly unphysiological conditions and was insignificant at any time point. We were also unable to see differences when assessing the susceptibility of either resting or preactivated T cells to apoptosis induction. Functional analysis of CTL and CD4^+^CD25^+^FoxP3^+^ T_reg_ cells *in vitro* did not reveal any impact of Ptch ablation on these cell types either. However, we cannot exclude that other lymphocyte populations such as induced T_reg_ cells might be affected by the absence of Ptch.

Since more subtle effects in T lymphocytes might only become evident *in vivo*, we used three models of adaptive immune responses addressing different aspects of T cell function. Nevertheless, we were unable to identify an impact of Ptch ablation in any of these settings.

Surprisingly, there was no evidence of activated canonical Hh signaling when we analyzed target gene expression in T cells from *Ptch^flox/flox^ CD4Cre^+/−^* mice. Both *Gli1* and *Gli2* levels were unchanged despite successful Ptch deletion. This is consistent with our previous data [12] and could be explained by the lack of primary cilia in cells of the hematopoietic lineage such as T cells [26]. Primary cilia are microtubule-based organelles that protrude from the surface of most vertebrate cells and fulfill crucial roles in vertebrate development by providing hubs for the transduction of various developmental signaling pathways including Wnt, FGF, PDGF and also Hh [27]. Although the interaction between Hh signaling and the primary cilium is currently not fully understood, it has recently become evident that the Hh pathway is strictly coupled to this cellular compartment [28]. Therefore, the lack of primary cilia may prevent Ptch-mediated activation of Hh signaling in T cells. This hypothesis is indirectly supported by work showing that the deletion of Ptch in hematopoietic stem cells also failed to activate canonic Hh signaling [10]. Alternatively, compensatory mechanisms might account for the canonical pathway not being activated by Ptch ablation. As a potential candidate mechanism, we investigated expression of the Ptch homolog Ptch2, which fulfills distinct roles from Ptch [29] but might still be able to compensate for its lack in selected cell types. However we were unable to detect *Ptch2* transcripts both in Ptch deficient and control T cells. Yet the existence of other compensatory mechanisms still has to be elucidated.

Previous studies by other groups had pursued different approaches to investigate the role of Hh signaling in T cells. Initial work focused on the *in vitro* response of human and murine (C57Bl/6) T cells to exogenous Shh. It was reported that addition of Shh enhanced T cell activation and proliferation induced by optimal or suboptimal concentrations of anti-CD3 and anti-CD28 antibodies whereas addition of an anti-Hh antibody to these cultures reduced activation and proliferation [3], [4], [13]. These changes were accompanied by an increased expression of the activation-dependent cell surface markers CD25 and CD69 and an enhanced secretion of cytokines such as IL-2 and IFNγ. Considering the current model of Hh signaling and our own findings, these results are difficult to explain. It is possible that addition of Shh to partially purified T cell populations activated these cells indirectly through other cell types present in the culture, or alternatively that Shh activated a non-canonical signaling pathway. It is also noteworthy that due to the artificial nature of *in vitro* experiments, the results may not reflect the physiological situation.

In another approach, Rowbotham and colleagues utilized transgenic mouse models on a C57Bl/6 background that either expressed the transcriptional activator (Gli2ΔN_2_) or repressor (Gli2ΔC_2_) form of Gli2 to study the effect of constitutive activation or repression of Hh signaling in T cells, respectively [8], [14]. They found that activation of the Hh signaling pathway exerted a negative impact on TCR signal strength with implications for positive and negative selection in the thymus and the function of peripheral T cells. Although the data obtained in both mouse models were consistent, the results need to be carefully evaluated in view of the design of the employed experimental system. Both models relied on the overexpression of artificial forms of the transcription factor Gli2 and due to this constraint, the obtained data does not necessarily reflect the physiologic function of this protein. Another group focused on the role of Hh signaling in T cells by inactivating the signal transducer Smo at different time points during T cell development [7]. They found that an early shutdown of Smo at the pro-T cell stage led to thymic atrophy, which was associated with a substantial decrease in thymocyte and peripheral T cell numbers. In contrast, a deletion of Smo in at a later stage of T cell development had no effect, neither on thymocyte numbers nor subtype distribution, suggesting that Hh signaling was essential for proliferation of early thymocytes while it becomes dispensable after pre-TCR expression. These findings are in line with the current model of Hh signaling as well as our own data. It is widely accepted that most vertebrate cells including stem cells possess primary cilia [30] while hematopoietic cells lose this compartment upon maturation. It is therefore conceivable that cells may also become refractory to Hh signaling once they are committed to the hematopoietic lineage. According to the findings of El Andaloussi et al. [7], the time window during which T cells lose responsiveness to Hh is the DN stage of thymocyte development. This suggests that canonical Hh signaling via Ptch is dispensable for all subsequent steps of T cell development as well as the function of peripheral T lymphocytes.

Collectively, our studies demonstrate that the Hh receptor Ptch is neither required for peripheral T cell function *in vitro* nor *in vivo*. We propose that this is either due to the lack of the primary cilium which is required for signal initiation in most cell types, or yet elusive compensatory mechanisms or a consequence of non-canonical Hh signaling. Therefore, it is unlikely that Ptch, but not necessarily Hh signaling itself, plays an essential role in peripheral T cell function and adaptive immunity.
